# The role of bile salts in liver regeneration

**DOI:** 10.1007/s12072-016-9723-8

**Published:** 2016-04-05

**Authors:** Liyanne F. M. van de Laarschot, Peter L. M. Jansen, Frank G. Schaap, Steven W. M. Olde Damink

**Affiliations:** Department of Surgery, NUTRIM School of Nutrition and Translational Research in Metabolism, Maastricht University, PO BOX 616, 6200 MD Maastricht, The Netherlands

**Keywords:** Bile salt homeostasis, Bile salt signaling, Farnesoid X receptor, Liver regeneration, Liver surgery

## Abstract

A growing body of evidence has demonstrated that bile salts are important for liver regeneration following partial hepatectomy. The relative bile salt overload after partial liver resection causes activation of bile salt receptors in non-parenchymal (viz. the plasma membrane receptor TGR5) and parenchymal (viz. the intracellular receptor FXR) cells in the liver, thus, providing signals to the regenerative process. Impaired bile salt signaling in mice with genetic deficiency of *Tgr5* or *Fxr* results in delayed liver regeneration after partial hepatectomy, and is accompanied by mortality in case of *Fxr* knock-out mice. Conversely, compensatory liver re-growth in hepatectomized mice can be stimulated by feeding of bile salts or alisol B 23-acetate, a natural triterpenoid agonist of Fxr. A large number of animal studies underscore the importance of strict maintenance of bile salt homeostasis for proper progression of liver regeneration. Both ileal and hepatic Fxr play a key role in regulation of bile salt homeostasis and, thus, preventing hepatotoxicity caused by excessive levels of bile salts. They further contribute to liver regeneration by induction of mitogenic factors. Agents that target bile salt receptors hold promise as drugs to stimulate liver regeneration in selected patients.

## Introduction

The liver has unmatched capacity for compensatory hyperplasia (‘regeneration’) after hepatic injury (e.g. toxic or inflammatory insults) or tissue loss. This feature allows segmental liver resections in patients with hepatobiliary tumors, as well as living-donor liver transplantation. Regeneration is also part of remodeling of the liver that occurs in cirrhosis, the replenishment of lost cells after hepatotoxic insults, and the transient hepatomegaly during pregnancy in response to the increased metabolic demand imposed by the developing fetus [1]. This review focuses on liver regeneration that occurs after surgical removal of liver mass. Partial hepatectomy (PHx) causes growth of the remnant liver until near restoration of its original size. This preservation of liver-to-body mass ratio (‘hepatostat’), and accordingly liver regeneration, is likely driven by one or more essential functions of the liver (‘metabolic demand’), as originally proposed by the late Nelson Fausto [2]. Bile salts are attractive candidates for such a metabolic signal as they are synthesized exclusively by the liver, with a major role of the liver in handling of these detergent-like molecules. Recognition of bile salts as signaling molecules and identification of dedicated bile salt receptors has boosted studies on the biological effects of bile salts. The emerging role of bile salt signaling in liver regeneration after PHx is discussed in this review.

## Liver regeneration after partial hepatectomy

A well-tolerated procedure for two-thirds PHx in rats was described by Higgins and Anderson in 1931, and forms the base of most studies on resection-induced liver regeneration [3]. The rodent liver is multi-lobed and surgical removal of three of the five lobes (approximately 70 % of the liver mass) leads to growth of the remnant lobes and almost full restoration of liver mass in 7–10 days. Rodent liver anatomy allows excision of these three lobes without causing damage—and attendant inflammation—to the two remaining lobes. Although inflammatory mediators play an important role in the regenerative process, the two-thirds PHx model is considered a clean model with no to minimal inflammation. This allows ‘clean’ dissection of the molecular events taking place during liver regeneration without interference of superimposed processes. In the clinical setting, liver resection typically involves dissection along segmental boundaries causing injury and inflammation in the remnant liver. This is also the case in rodent models of hepatotoxin-induced liver regeneration like the carbon tetrachloride model, where an inflammatory response results in removal of necrotic/apoptotic hepatocytes prior to replenishment of lost cells. In mice subjected to two-thirds PHx, peak DNA synthesis in hepatocytes is observed between 36 and 48 h. Earlier restoration of initial liver mass, as reflected by a shift towards an earlier time point of this peak or a greater proportion of hepatocytes in S phase at peak time, is referred to in this review as accelerated liver regeneration. Conversely, impaired regeneration is reflected by shifts in the opposite direction and results in delayed recovery of liver mass.

PHx can be further extended to removal of 90 % of the liver mass but this causes considerable mortality due to small-for-size syndrome and subsequent post-resectional liver failure (PLF) [4]. The model of extended PHx is used to study PLF, a dreaded complication of liver surgery. Depending on the quality of the liver parenchyma, a future remnant liver volume (FRLV) of 25–40 % of the estimated total liver volume is regarded as the minimum to safely undergo PHx. Patients with insufficient FRLV can undergo pre-operative portal vein embolization (PVE) to prevent complications following PHx [5]. PVE with or without staged hepatectomy uses the regenerative capacity of the liver to enlarge the FRLV, and enables surgical resection in patients with initially non-resectable tumors. Occlusion of the portal vein branches supplying the tumor-bearing segments results in atrophy of these segments and compensatory growth of the contralateral segments. With this technique the size of the FRLV can be increased up to 62 % of the original FRLV [5, 6].

## Molecular events after partial hepatectomy

Liver regeneration has been studied scarcely in humans, and our knowledge of the underlying molecular events is largely based on findings from animal experiments. Excellent reviews covering the successive phases in liver regeneration in-depth have been published elsewhere [7, 8]. In short, compensatory liver growth after surgical resection does not require stem cells or progenitor cells, but involves replication of existing mature liver cells [7]. Two-thirds PHx results in increases in portal and sinusoidal blood flow through the remnant liver. The combination of shear stress-activated pathways, extracellular matrix remodeling with release of matrix-bound growth factors, and a relative increase in supply of signaling molecules from the (portal) circulation, initiate the regenerative cascade [7, 9]. Hypertrophy of hepatocytes in the remnant liver is a first and immediate event after PHx [8, 10, 11]. Within 30 min after PHx, intrahepatic levels of tumor necrosis factor alpha and interleukin-6 increase and signaling via their respective receptors causes activation of the transcription factors nuclear factor-kappa B and signal transducer and activator of transcription 3 (STAT3) [7, 8]. This causes quiescent hepatocytes (G_0_ phase) to re-enter the cell cycle [12]. This priming of hepatocytes is necessary to sensitize the cells to growth factors that drive subsequent cell cycle progression [2, 13]. After PHx, hepatocytes rapidly divide once or twice before returning to proliferative quiescence [7]. The systemic level of signaling molecules, such as hepatocyte growth factor, increases after PHx and this contributes to the initiation of DNA synthesis [9]. An increase in the same signaling molecules is also found in the peripheral circulation of healthy donors undergoing right hepatectomy for living-donor liver transplantation [14]. The presence of hepatocyte growth factor and epidermal growth factor receptor ligands is necessary for further progression of hepatocytes through the cell cycle [8]. The onset of hepatocyte DNA synthesis begins in the periportal region and proceeds pericentrally [7]. Hepatocytes provide the mitogens that induce proliferation of the non-parenchymal cells [15]. Of all liver cells, (periportal) hepatocytes replicate first, followed by division of non-parenchymal cells such as cholangiocytes, sinusoidal endothelial cells and Kupffer cells [7]. A small wave of apoptosis reduces the number of hepatocytes at the end of the regenerative process, suggesting that the number of produced hepatocytes may have exceeded the original number. Little is known about the signaling events involved in termination of the regenerative process, but signaling via transforming growth factor β1 has been implicated [8, 9, 16]. Suppression of hepatocyte proliferation may involve regulatory RNAs, including miR34a which is highly upregulated in the late phase of liver regeneration, and their yet-to-be-defined targets [17]. As discussed in more detail below, bile salt signaling via endocrine fibroblast growth factor 19 (FGF19) has been proposed to regulate final liver size [18].

## Bile salt signaling

**Fig. 1:**
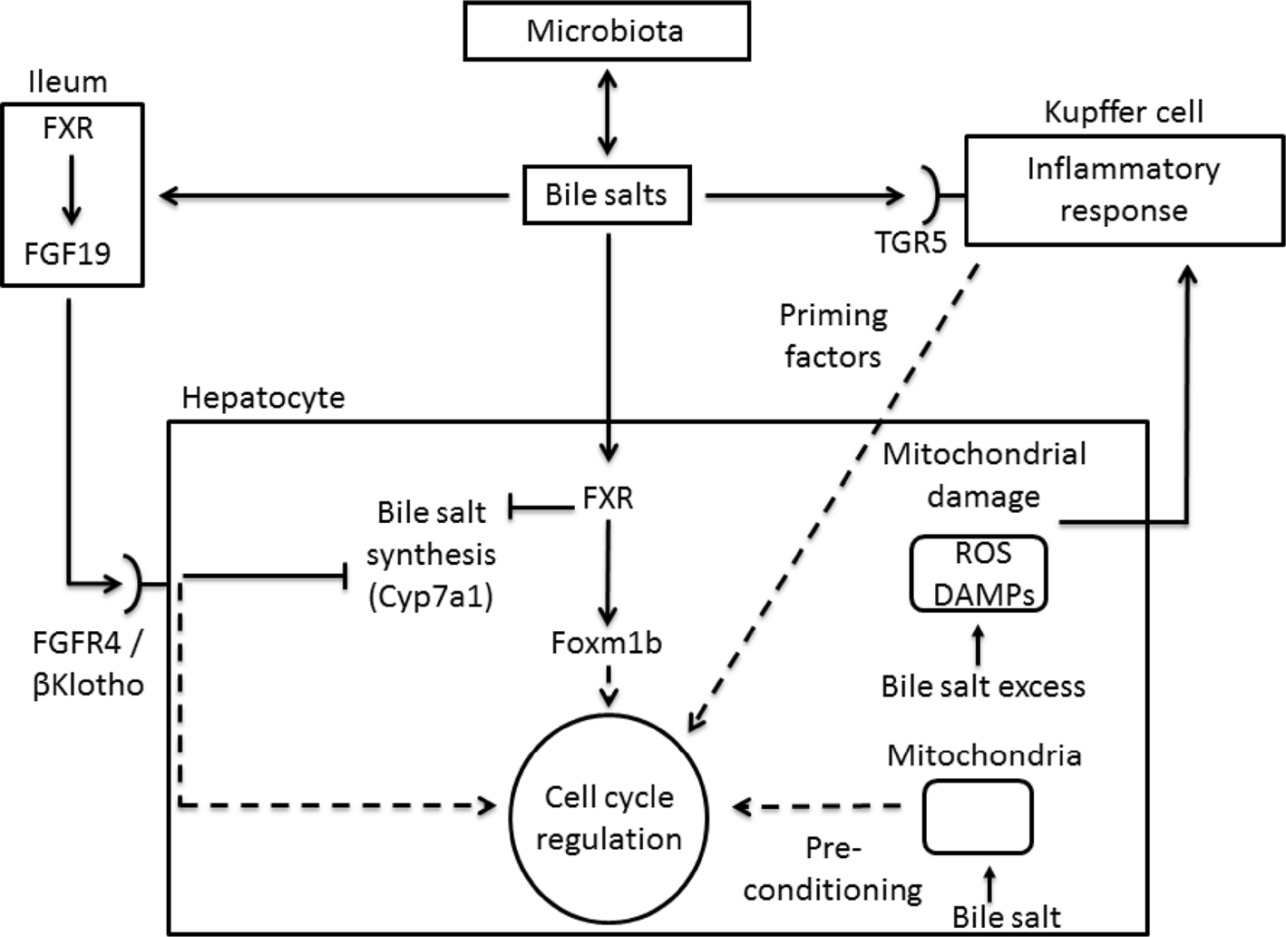
Emerging roles of bile salts in liver regeneration after partial hepatectomy. Circulating and hepatic levels of bile salts rise shortly after PHx. This causes activation of bile salt receptors at the cell surface of Kupffer cells (TGR5) and inside the hepatocyte (FXR). Kupffer cells release soluble factors that prime cell cycle re-entry of quiescent hepatocytes. FXR regulates cell cycle progression through induction of Foxm1b, and through the ileal FXR/FGF19/FGFR4 signaling axis. Bile salt levels in the hepatocyte need to be tightly controlled to prevent toxicity. Excessive bile salt levels result in mitochondrial damage and release of reactive oxygen species (ROS) and damage-associated molecular patterns (DAMPs) that can trigger activation of nearby Kupffer cells. An exaggerated inflammatory response of Kupffer cells results in apoptosis and necrosis of hepatocytes. Slightly elevated bile salt levels may stimulate cellular antioxidant defense responses and precondition the liver. FXR and signaling via FGF19/FGFR4/βKlotho play an important role in bile salt homeostasis, amongst others by exerting negative feedback control of bile salt synthesis. The composition, and hence the signaling properties, of the circulating bile salt pool is determined by the gut flora. The influence of the gut microbiome on liver regeneration after PHx is being explored

Bile salts are the major end products of cholesterol catabolism. They are synthesized exclusively by the liver, and maintained as an enterohepatic cycling pool [19]. Besides their classical role in dietary lipid utilization, bile salts act as (postprandial) signaling molecules that activate dedicated receptors at the cell surface (i.e. TGR5) and inside the cell (e.g. Farnesoid X Receptor; FXR) [20, 21]. Because bile salts are detergents, they can damage intracellular membranes (i.e. mitochondria) and trigger apoptosis or necrosis of hepatocytes [22]. The detrimental effect on mitochondrial integrity is brought about by hydrophobic bile salts in particular, and results in generation of reactive oxygen species that may further aggravate hepatocyte injury by activating nearby Kupffer cells [22]. On the other hand, levels of bile salts below a certain threshold appear to promote anti-oxidant defenses and may in fact pre-condition the liver and have a stimulatory effect on liver regeneration [23, 24] (Fig. 1). The intracellular bile salt receptor FXR plays a key role in maintaining intrahepatic bile salt levels within safe limits, and thus preventing toxic consequences of bile salt overload. FXR controls bile salt homeostasis by coordinating synthesis, uptake, conjugation and secretion of bile salts. Regulation of bile salt synthesis occurs primarily at the level of cholesterol-7α-hydroxylase (*CYP7A1)* transcription and involves FXR expressed in the terminal ileum and the liver [25] (Fig. 1). This pathway will be explained in more detail below.

## Bile salts and liver regeneration

Bile salt signaling has emerged as an important player in liver regeneration after liver resection [26]. In a pioneering study of Huang et al. it was demonstrated that bile salt feeding (viz. cholic acid, a hydrophilic bile salt) induced hepatomegaly in mice with an intact and non-injured liver [26]. Although a bile salt overload can trigger a proliferative response by causing hepatic injury, a cholic acid diet did not induce substantial toxic effects with a subsequent regenerative response. A moderate bile salt overload thus appears to act as a regenerative trigger per se [1, 26, 27]. Dietary bile salt-supplementation also accelerated liver regeneration after PHx, an effect that depended on the presence of Fxr [26]. Conversely, depletion of hepatic bile salts by a bile salt-sequestering resin resulted in impaired DNA synthesis and liver regrowth [26, 28]. In bile salt-deficient *Cyp27a1*^−*/*−^ mice, liver regeneration after PHx was impaired [29]. Liver growth did not occur in the first 40 h after PHx and DNA synthesis and mitosis was reduced [29]. Likewise, disturbed hepatocyte proliferation and liver regrowth was observed in hepatectomized rats in which the bile salt pool was depleted via a biliary fistula. Intestinal infusion of taurocholate could reverse the defective regeneration in this model [30]. The findings from above gain- and loss-of-function models stress the importance of bile salt signaling, and by extension an intact enterohepatic circulation, for efficient regeneration of the liver after PHx.

Following PHx in rodents, an increase in systemic bile salts is detectable already after 1 min and levels reach a peak during the first 24 h [26, 31–34]. This rapid incline is likely due to hemodynamic alterations exposing the remnant liver to a relative bile salt overload [33]. The systemic elevation of bile salts can result in signaling via TGR5, which is expressed at the cell surface of Kupffer cells. Increased bile salt content of the hepatic remnant is apparent after 1 h, and this allows activation of hepatocytic Fxr. The elevations of circulating and hepatic bile salts are transient and normalize approximately 2 days after 70 % PHx in mice [25].

After non-surgical reduction of functional liver mass by PVE, serum bile salts also increase and this correlates with the regenerative response in rabbits [35]. When portal vein ligation is combined with segmental bile duct ligation in rats, atrophy of the ligated segments and hypertrophy of the contralateral segment is augmented in comparison with portal vein ligation only. Both effects depended on enhanced bile salt retention upon bile duct ligation. This drove enhanced apoptosis in the ligated segments while stimulating proliferation of the non-ligated segment [36]. Bile salt signaling may play a role in human liver regeneration as well. External biliary drainage in patients undergoing hemihepatectomy resulted in lowering of systemic bile salt levels and reduced liver regrowth after resection [37]. Furthermore, increased systemic bile salt levels were associated with regeneration in patients undergoing PVE as a preoperative procedure [38].

Although low bile salt levels impair liver regeneration, an intrahepatic bile salt overload causes hepatotoxic effects [39]. While diet containing 0.2 % cholic acid is stimulatory, feeding of a 1.0 % cholic acid diet resulted in mortality in hepatectomized mice indicating that toxic bile salt levels had been reached [40]. PHx in mice is accompanied by decreased basolateral uptake and synthesis of bile salts, while bile salt secretion is increased [41]. Fxr-dependent downregulation of *Cyp7a1* accounts for decreased bile salt synthesis in mice after PHx [40]. When *Cyp7a1* is not suppressed due to genetic *Fxr* deficiency or transgenic overexpression of *CYP7A1*, liver regeneration is impaired by outbalanced apoptosis and decreased DNA synthesis resulting in reduced post-PHx survival [40]. Above notions stress the importance of strict maintenance of intrahepatic bile salt homeostasis for proper progression of liver regeneration.

## Farnesoid X receptor and liver regeneration

FXR is expressed at high levels in the liver and the distal small intestine [25]. The primary bile salt chenodeoxycholic acid is its most potent endogenous ligand [25]. Both ileal and hepatic Fxr are engaged in negative feedback regulation of bile salt synthesis by bile salts. Binding of bile salts to ileal FXR results in the induction of *Fgf15/FGF19* (fibroblast growth factor) expression. Fgf15/FGF19 is an endocrine-acting factor that is released in the portal circulation. Binding of Fgf15/FGF19 to its hepatic receptor (complex of Fgfr4 and βKlotho) results in activation of a signaling cascade that causes downregulation of *Cyp7a1* and diminished bile salt synthesis [42–44] (Fig. 1). Activation of hepatic Fxr by bile salts results in the induction of *Shp*, encoding a transcriptional repressor that targets *Cyp7a1* thus reducing bile salt synthesis.

Bile salt homeostasis is dysregulated in *Fxr*^−*/*−^ mice, and PHx in these mice results in delayed liver regeneration and mortality, and loss of the regeneration-stimulating effect of a 0.2 % cholic acid diet [26]. Impaired activation of Stat3 and delayed initiation of DNA replication have been implicated in the defective regeneration in hepatectomized *Fxr*^−*/*−^ mice [45]. Moreover, Fxr can directly activate Forkhead box m1b (*Foxm1b*), an injury-induced transcription factor that is required for cell cycle progression [46] (Fig. 1). Although liver regeneration following PHx was delayed in mice with liver-specific deletion of *Fxr*, these mice showed less severe outcomes after PHx than mice with global deficiency of *Fxr* [47, 48]. This indicates that Fxr outside the liver participates in liver regeneration. Defective liver regeneration after PHx was also apparent in mice with intestine-specific deletion of *Fxr,* with adenoviral *Fgf15* delivery able to overcome this defect [48]. Both intestinal and liver Fxr are required for normal liver regeneration after PHx, thus, ensuring maintained bile salt homeostasis and appropriate regulation of genes engaged in proliferation, e.g. *Foxm1b.*

Fgf15 appears to serve a double role in liver regeneration through effects on bile salt homeostasis and by acting as a mitogen for hepatocytes and cholangioytes [25]. PHx in *Fgf15* knockout mice results in higher mortality than in mice lacking *Fxr* [26, 32, 49]. The hepatic expression of the Fgf15/FGF19 receptor Fgfr4 increases after PHx [50]. Mice lacking *Fgfr4* show increased mortality and severe liver necrosis after PHx, along with increased *Cyp7a1* expression and increased hepatic bile salt content [51]. Reduced activation of Stat3 and lowered expression of *Foxm1b* likely participate in defective liver regeneration. The liver-to-body weight ratio was only mildly reduced in hepatectomized *Fgfr4*^−*/*−^ mice as a result of cellular hypertrophy that compensated reduced hyperplasia [51]. The survival of mice after extended liver resection (85 % PHx, a surgical model for acute liver failure) can be improved by exogenous administration of Fgf15 [32].

Apart from involvement in the initial phases of liver regeneration, bile salt/FGF19 signaling may play a broader role in regulation of liver mass. Cessation of FGF19 signaling after the liver-to-body mass ratio approximates pre-PHx values may be involved in the termination of liver regeneration. In an elegant study, it was demonstrated that repopulation of immune deficient mice (FRG model) with human hepatocytes resulted in hepatomegaly and near doubling of liver-to-body mass ratio [17]. This effect was attributed to expansion of the bile salt pool due to unopposed bile salt synthesis in transplanted human hepatocytes, which are refractory to the bile salt synthesis-repressing effect of endogenous Fgf15 (the rodent equivalent of human FGF19). Bile salt homeostasis and liver-to-body mass ratio were normalized in human hepatocyte-repopulated mice expressing a transgene containing the *FGF19* gene with flanking regulatory regions. This allowed physiological induction of *FGF19* by bile salts, initiating a negative feedback response to suppress bile salt synthesis. The above findings are consistent with a model in which liver growth occurs when the bile salt pool exceeds the hepatic capacity to handle the load, and ceases upon reaching sufficient handling capacity. In line with such notion, a higher liver-to-body weight ratio is found in intestine-specific *Fxr* knockout mice, which exhibit reduced levels of *Fgf15*, elevated *Cyp7a1* expression and an enlarged bile salt pool [52].

## TGR5 and liver regeneration

TGR5 is a plasma membrane receptor for bile salts, showing the greatest affinity for secondary bile salts [25, 53]. It is widely distributed throughout the gastrointestinal tract and exerts multiple functions in energy homeostasis and inflammation. In the liver, Tgr5 is expressed by Kupffer cells and cholangiocytes. PHx in *Tgr5*^−*/*−^ mice resulted in prolonged elevations of circulating and hepatic bile salts, severe necrosis, an aggravated inflammatory response, and delayed liver regeneration [54]. The liver injury observed in hepatectomized *Tgr5*^−*/*−^ mice is likely caused by bile salt-induced toxicity [55]. Thus, although the mechanisms are incompletely understood, Tgr5 appears to be important for protecting the remnant liver against the hepatotoxicity related to the transient bile salt overload after PHx.

## The interplay between gut microbiota and bile salts during liver regeneration

The gut microbiota play an important role in cell proliferation following PHx by the action of bacterial endotoxins on cells of the liver’s innate immune system, which serves a crucial role in priming hepatocellular cell cycle re-entry [56]. Moreover, the gut microbiota may act indirectly by affecting the composition, and hence signaling properties, of the circulating bile salt pool. Certain microbial species in the colon are equipped with enzymes that convert the host’s primary bile salt species into secondary bile salts, thus, altering their affinity for TGR5 and FXR. During liver regeneration following PHx, the composition of the gut microbiome changes [57]. A direct correlation was found between the concentration of the different bile salts, expression of genes involved in bile salt homeostasis *Shp* and *Cyp7a1,* and the gut microbiota composition [57]. It will be interesting to gain further insights how the microbiota-bile salt interaction influences liver regeneration, and whether a probiotic approach can precondition the liver prior to liver surgery.

## Pharmacological modulation of liver regeneration by bile salt receptor agonism

Data from animal studies indicate that FXR agonists have therapeutic potential to accelerate liver regeneration after PHx. Cholic acid feeding augmented liver regeneration following PHx in Fxr-dependent manner [26]. Dose-dependent stimulation of liver regeneration was also observed in mice given alisol B 23-acetate, a plant triterpenoid with FXR agonistic activity [58]. Lastly, the synthetic FXR agonist Px20350 could overcome defective regeneration in aged mice [39]. In a clinical context, impaired regeneration of the (small and/or compromised) remnant liver can result in PLF. Cholestasis is an established risk factor for PLF [59], and patients with jaundice due to bile duct obstruction or parenchymal liver disease have increased morbidity rates following PHx [59, 60]. This implicates dysregulated bile salt homeostasis and bile salt toxicity in the defective regenerative response observed in patients with PLF, as mirrored in impaired liver regeneration in *Fxr* and *Tgr5* knockout models [26, 47, 48, 54, 55]. Enhanced Kupffer cell activation is thought to occur in PLF, resulting in an excessive inflammatory response and hepatocyte death through pro-inflammatory cytokine triggered pathways [60]. Bile salt toxicity may contribute to the hyperactivation of Kupffer cells in the context of PLF, by release of damage signals from injured hepatocytes. It will be interesting to explore whether FXR/FGF19 (improved bile salt homeostasis, induction of pro-regenerative factors) and/or TGR5 (dampening of inflammatory response in Kupffer cells) agonism is useful in the management of PLF [61].

## Conclusion and future directions

Bile salts have emerged as important players in liver regeneration following PHx. FXR and TGR5 are the main mediators of the actions of bile salts. FXR plays a key role in maintaining bile salt homeostasis, a prerequisite for normal progression of liver regeneration. FXR also controls the expression of *Foxm1b*, a transcription factor with a crucial function in cell cycle progression. TGR5 protects the liver during the transient bile salt overload after PHx, likely by preventing an excessive inflammatory response to toxic bile salts. A contribution of the gut microbiota in modulation of liver regeneration is emerging, and this may involve effects via bile salt signaling [56, 57]. Certain microbial species can convert the host’s primary bile salts to secondary bile salt species, and accordingly influence the affinity for bile salt binding to FXR or TGR5. Apart from potential modulation by gut microbial composition, FXR and TGR5 are both amenable to pharmaceutical targeting. Animal studies indicate that Fxr agonism can accelerate liver regeneration after PHx, while the FXR–regulated enterokine FGF19 can reduce mortality in a surgical model of acute liver failure. It is worthwhile to explore these avenues for the treatment of clinical conditions that are caused by insufficient liver regeneration, such as post-resectional liver failure.
